# Supplementary material for: Identification of photoperiod-induced specific miRNAs in the adrenal glands of Sunite sheep (Ovis aries)
Source: Front Vet Sci. 2022 Jul 22;9:888207. doi: 10.3389/fvets.2022.888207 (PMC9354845; doi:10.3389/fvets.2022.888207)
Supplement: Supplementary file 1 [file Data_Sheet_1.ZIP › Supplementary file/miRDeep2 file.zip!/miRDeep2 file/Know_Structure/LP42A1/oar-mir-329a.pdf]

miRBase precursor : oar-mir-329a
Total read count : 45
oar-miR-329a-5p read count : 4
oar-miR-329a-3p read count : 41
remaining reads : 0

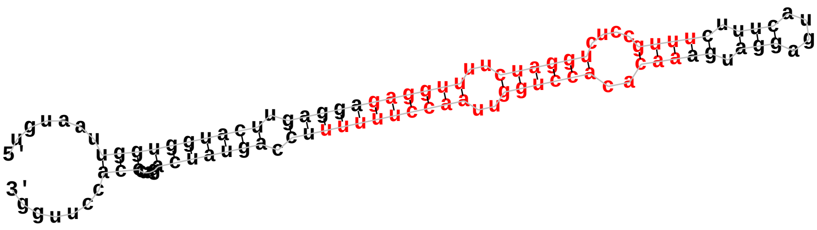

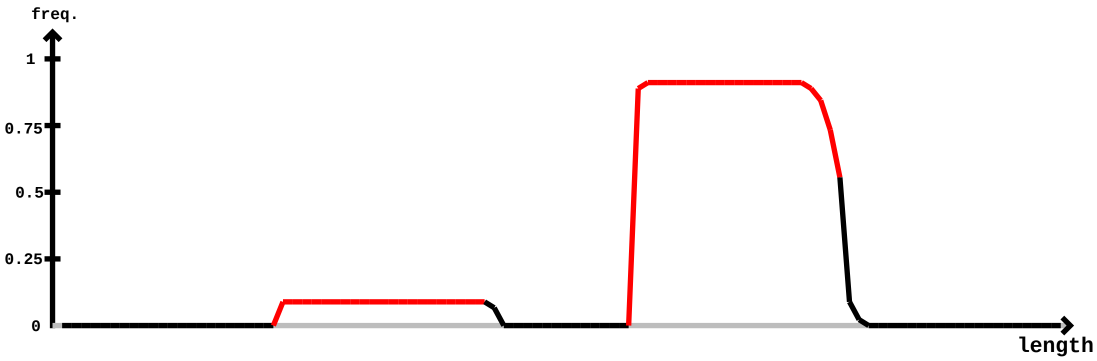

oar-miR-329a-5p

oar-miR-329a-3p

```
5'- uguaauuggugguacuugaggagagguuuucuaggucuccguuucuuucaugaggaugaaacacaccugguuaaccuuuuuuccaguaucagaucccaccuugg   -3'   exp
    .......(((((((((.(((((((((((..((((((....(((((.(((....))).)))))..))))))..))))))))))).)))).......)))))....   reads   mm   sample
    ......................gagguuuucuaggucuccguuu............................................................   1       0    seq
    ......................gagguuuucuaggucuccguuuc...........................................................   3       0    seq
    ...........................................................aacacaccugguuaaccu..........................   1       0    seq
    ...........................................................aacacaccugguuaaccuu.........................   2       0    seq
    ...........................................................aacacaccugguuaaccuuu........................   5       0    seq
    ...........................................................aacacaccugguuaaccuuuu.......................   8       0    seq
    ...........................................................aacacaccugguuaaccuuuuu......................   21      0    seq
    ...........................................................aacacaccugguuaaccuuuuuu.....................   3       0    seq
    ............................................................acacaccugguuaaccuuuuuuc.....................   1       0    seq
```
